# 13.4 CANNABINOID RECEPTOR GENE POLYMORPHISMS AND COGNITIVE PERFORMANCE IN PATIENTS WITH SCHIZOPHRENIA

**DOI:** 10.1093/schbul/sby014.052

**Published:** 2018-04-01

**Authors:** Joao Salgado, Rodrigo Ferretjans, Bruna Panizzutti, Eduarda Rosa, Clarissa Gama

**Affiliations:** 1 Federal University of Minas Gerais; 2 Federal University of Rio Grande do Sul

## Abstract

**Background:**

Cognition is a major determinant of functioning in patients with schizophrenia. There is evidence that the endocannabinoid system influences cognition in human subjects, and participates in the pathophysiology of schizophrenia. In a previous study, we have shown that the expression of cannabinoid receptors (CB1R and CB2R) on peripheral lymphocytes is inversely correlated with performance in the Brief Assessment of Cognition in Schizophrenia (BACS), in patients with schizophrenia. Recently, CBRs polymorphisms have been associated with an increased risk for schizophrenia, structural changes in the central nervous systems and in cognitive performance of the patients. The aim of the present study was to investigate the association between CBRs polymorphisms and cognitive performance as assessed by the BACS.

**Methods:**

A sample of 85 stable medicated patients (61% men; age = 41.6 ± 12.2 years; illness duration = 12.8 ± 10.7 years) was enrolled in this study. Two CB1R polymorphisms (rs1049353; rs12720071) and one CB2R polymorphism (rs2229579) were tested.

**Results:**

We did not find any difference in general cognitive performance (BACS total score) regarding the three polymorphisms tested. However, when we analysed specific cognitive domains we have found a significant difference (p=0.002) regarding working memory (assessed by the Digit Span test) in patients with the rs12720071 polymorphism, where those with allele C performed better than those with T/T genotype. Since about a third of the patients (34%) had a history of past use of cannabis and 2.5% reported current use, we performed the rs12720071 polymorphism analysis excluding these patients. In this subgroup of patients, those with allele C also performed significantly better on Digit Span test (p=0.037).

**Discussion:**

In this sample, the rs12720071 polymorphism of CB1R appears to influence performance on a working memory task that is sensitive to prefrontal cortex function.

